# The gender pay gap is smaller in occupations with a higher ratio of men: Evidence from a national panel study

**DOI:** 10.1371/journal.pone.0270343

**Published:** 2022-07-06

**Authors:** Sarah Schneider, Katrin Rentzsch, Astrid Schütz

**Affiliations:** 1 Department of Psychology, University of Bamberg, Bamberg, Germany; 2 Department of Psychology, Psychologische Hochschule Berlin, Berlin, Germany; FAME|GRAPE, POLAND

## Abstract

Gender differences in career success are still an issue in society and research, and men typically earn higher incomes than women do. Building on previous theorizing and findings with the Theory of Gendered Organizations and the Theory of Tokenism, we used a large sample of the adult starting cohort in the German National Educational Panel Study and a multilevel approach to test how the interaction between gender and the gender ratio in occupations was associated with income. We wanted to know whether the male advantage in terms of income would be equal in magnitude across occupations (as suggested by the Theory of Gendered Organizations) or if it would vary with the gender ratio in occupations (as suggested by the Theory of Tokenism and reasoning regarding person-job fit), such that people benefit either (a) from resembling the majority of employees in a field by working in a gender-typical occupation or (b) from standing out by working in a gender-atypical occupation. Analyses supported the hypothesis that employees’ incomes may benefit if they belong to the gender minority in an occupation, but this finding applied only to women. By contrast, men did not benefit from working in a gender-atypical occupation. Thus, women earned less than men earned overall, but the gender pay gap was smaller in occupations with a higher ratio of male employees. The findings can advance the understanding of gender-related career decisions for both employers and employees.

## Introduction

### Explanations for the gender pay gap: Gendered organizations

The Equal Pay Day marks the annual difference in wages between men and women by identifying how far into the year (e.g., March 07, 2023) women must work for free (so to speak) in order to catch up with what men had earned in the previous year [1]. Even though men in Western societies drop out of school more often and are less likely to receive a university degree than women [2,3], there is still a clear male advantage regarding income in various countries [2,4–10], and as the participation of women in the labor market increases, the gender pay gap even increases [11]. As Noll and Bachmann [12] satirically suggested, if you want to be successful in your career, you better not be a woman.

Several studies and meta-analyses have shown that, overall, men have higher incomes than women do [2,9]. Such income differences are partly explained by the facts that women more often work part time and less often occupy leadership positions than men [13–16].

At the same time, the proportions of men and women in different types of occupations vary (labor market segregation) [17–21]–and these occupations also vary with respect to pay [17,21]. For example, men more often choose occupations that are competitive or require physical power [22], such as management positions or occupations in production and construction, whereas women can more often be found in less competitive occupations and in the social sector [22], such as occupations in care and nursing.

According to data from the Federal Labour Office, Germany [Bundesagentur für Arbeit] [23], people in occupations with a higher ratio of men tend to receive better pay than those in occupations with a higher ratio of women. Occupations with a higher ratio of men include the highest paying fields, such as STEM occupations as well as jobs on executive boards [24–28]. Occupations in which women are overrepresented often offer lower wages [29] (see also the status composition hypothesis [30]) and are overall less prestigious than occupations with a higher ratio of men [31].

How the male advantage can be observed across the labor market has been investigated on the basis of the Theory of Gendered Organizations [32,33], which states that men are advantaged because gender stereotypes that favor men are well established in the working world. Women’s opportunities are often limited because they are often not perceived as representing the characteristics of an ideal worker [18]. For example, the lack-of-fit framework [34,35] suggests that discrimination can result from stereotypical beliefs about the prevalence of communal traits in women and agentic traits in men and the overall more positive evaluation of agentic traits for the labor market [2,36,37]. Analyzing data from 1979 to 1993 in a sample of career starters, and differentiating between three categories of occupations (predominantly male, neutral, and predominantly female), the results of a study using U.S. data from the National Longitudinal Survey of Youth showed a main effect of employees’ gender on income independent of the gender ratio in an occupation [18]. Results were interpreted to mean that the male advantage is equal in magnitude across occupations (as stated by the Theory of Gendered Organizations [32,33]).

To test whether the Theory of Gendered Organizations would be supported in a recent German national sample of employees, we advance the hypothesis that the male advantage in income can be found across occupations, irrespective of their gender ratio:

H1: *The male advantage in income is equal in magnitude across occupations*.

### Working in gender-atypical or gender-typical occupations–which is more advantageous from a financial perspective?

Whereas the Theory of Gendered Organizations [19,32] and previous results [18] suggest that the male advantage in income is independent of the gender ratio in occupations, other research suggests that the gender pay gap varies between occupations and that the gender ratio in the occupation is a relevant factor. If the gender ratio makes a difference, there are two possibilities for the direction of the association. It could be advantageous for employees to work in gender-typical occupations, or the contrary could be true. Both assumptions have been supported by theory and previous findings as elaborated on below. With this study, we aimed to test these assumptions against each other.

On the one hand, it has been shown that the pay employees receive is partly based on the extent to which their abilities match the demands of an occupation and the expectations of their work environment and employer [38,39]. Thus, employees working in a gender-typical occupation may be rewarded for fitting in, even more so as employment conditions, the work environment, and last but not least, the wage structure are to a large extent regulated by employers and their attitudes [40]. This association is also supported by Social Role Theory [41,42]: Violations of gender-based expectations are often punished and may lead to social and economic disadvantages [43–45]. Employees who fulfill gender-based expectations by working in gender-typical occupations should thus earn more than those who violate gender-based expectations by working in gender-atypical occupations.

Furthermore, it may pay off to work in an occupation where one is seemingly “invisible” because one belongs to the gender majority. The term “invisibility” here describes the adaptation to the social standards in an occupation [46]. According to Lewis and Simpson [47], invisibility provides a source of power because particularly those employees who belong to the majority have the power to establish standards for work skills and performance in an occupation. Because such employees typically comply with the standards they have established, they have the chance to ascend the hierarchy quickly. By contrast, members of the minority face difficulties because they have to adjust to the majority’s norms and expectations. According to this view, merely belonging to the gender majority would promote career success [48].

Other research that has built on the Theory of Tokenism [49] has implied that incomes might be higher for people who work in occupations in which they belong to the minority. Whereas Kanter [49] argued that being a token could have negative effects on one’s career advancement, later research on tokenism in the workplace did not support this assumption. For example, men who worked in occupations dominated by women, such as nursing, received higher recognition [50,51] and were more likely to be associated with the prestigious group of physicians [52]. It has been argued that individuals who work in a gender-atypical occupation enjoy an “exotic” status, as they are more noticeable. For example, men may be seen as “exotic” when they choose a predominantly female occupation, such as nurse [52–54] or flight attendant [55]. Such employees might be more visible to employers and may thus have a higher chance to be singled out for bonuses or promotions [56]. Indeed, research has shown that male nurses often ascend the hierarchy more quickly than their female colleagues and more often occupy better paying leadership positions [53]. In these cases, gender-related visibility adds to men’s overall advantage. Furthermore, research has shown that men working in female-dominated occupations tend to highlight their gender-specific strengths [54,57], which further contributes to their visibility [56].

However, there seem to be gender differences in how gender-related visibility is associated with advantages in the working world [46,51,56,57]. Whereas men working in gender-atypical fields often experience a great deal of support from their work environment [4,58] which may add to their overall income advantage, women in gender-atypical occupations often face high performance pressure and career barriers [47]. Although gender-related visibility may add to an overall advantage for men in the case of predominantly female occupations and the general increase in demands for gender diversity [59,60], it is possible that the systematic disadvantage of predominantly female occupations [30,60] will level out the advantage of gender-related visibility or gender diversity for men in female-dominated occupations. Even though they still face career barriers in predominantly male occupations [47], for women, gender-related visibility may narrow the gender pay gap in occupations that have recently faced increases in demands for gender diversity, such as male-dominated STEM occupations [61].

Furthermore, the increased focus on gender diversity in hiring processes could entail possible income advantages for women if they are underrepresented in an occupation [62,63]. Recent representative data has shown for Western [62] and Eastern work cultures [61] that the rising demand for gender diversity in occupations has reduced gender disparities in both male-dominated STEM fields and management jobs. For example, multiple simulation studies with representative data from the United States showed that women who applied for positions in male-dominated fields such as STEM sciences were preferred to men in a simulated hiring process [62]. Furthermore, national panel data from Japan showed an increase in the representation of women on executive boards across various sectors [61].

As elaborated on above, in considering the arguments that there may be an income advantage due to belonging to either the gender majority (i.e., gender-related fit) or the gender minority (i.e., gender-related visibility) in an occupation, we propose the following competing hypotheses.

H2a: *The pay gap between women and men should be larger in occupations with a higher ratio of men than in occupations with a lower ratio of men*.

H2b: *The pay gap between women and men should be smaller in occupations with a higher ratio of men than in occupations with a lower ratio of men*.

### The present study

Building on the established findings of the male advantage (or the negative association between gender and income [9]) as suggested by the Theory of Gendered Organizations and the positive association of the ratio of men to women in occupations [24] and income, the main goal of the present study was to test whether the male advantage in terms of income was equal in magnitude across occupations or if it varied with occupational gender ratios. In doing so, we wanted to test hypotheses on the magnitude of the male income advantage and whether it would vary across occupations with different gender ratios. If the male advantage in income were to vary across occupations, we aimed to further analyze the direction of that association [64] because a positive interaction between gender and the gender ratio would imply that an increasing ratio of men in occupations narrows the gender pay gap by reducing the male advantage. By contrast, a negative interaction would imply an increase in the gender pay gap with an increasing ratio of men in occupations.

We tested the present hypotheses in a nationally representative sample of German employees by using multilevel random coefficient modeling (MRCM [65]). Instead of using a categorical operationalization of occupations [18], we used a more fine-grained approach and included the gender ratio as a continuous variable.

## Materials and methods

### Participants

We used data from the cohort of adults from the National Educational Panel Survey (NEPS) conducted by the Leibniz Institute for Educational Trajectories. The German National Educational Panel Study assesses and describes educational processes and trajectories across the entire life span, as well as the consequences of education for life courses. The study follows a multicohort sequence design by differentiating and assessing data from six cohorts (early childhood, Kindergarten children, 5th graders, 9th graders, first-year college students, and adults) drawn from representative samples of the German population [66]. The panel study collects cohort-specific data, therefore in the adult cohort also includes various items on employment. The data we analyzed came from the scientific use file (SUF version 8.0.0; [67]). For the present analyses, we combined all data from participants from the Basic data set (regarding age and gender, no wave structure), the Competence data set from Wave 7 (2014/2015), and the Employment data set from Wave 8 (2015/2016). After combining the entries from the three data sets, the total sample size was *N* = 7,537 with an average age of 50.98 years (*SD* = 9.71) including 3,837 male and 3,700 female participants.

We excluded participants whose incomes were outliers or seemed implausible (e.g., income that deviated heavily from plausible incomes, such as a monthly gross income for a nurse of 15,000€ or an income of zero for employed participants). In order to detect outliers in income, we followed the upper-outer-fence method [68] by adding three times the interquartile range to the upper quartile in income for a cut-off value. Consequently, participants with more than 12,500€ monthly gross income were excluded from the analyses. On the basis of these criteria, a total of 567 participants were excluded from the analyses, resulting in *N* = 6,070 participants (3,085 men; 2,985 women) with an average age of *M* = 51.21 years (*SD* = 9.66) who were included in the analyses. The average monthly income of participants was 3,158.76 € (*SD* = 2046.07). A total of 439 men (14.23%) reported working part-time jobs, 2,635 men (85.41%) had full-time jobs, whereas 1,790 women (59.97%) had part-time jobs, and 1,182 women (39.60%) had full-time jobs. With respect to leadership positions, 944 men (30.6%) and 487 women (16.31%) occupied a leadership position.

### Measures

*Age* and *gender* were collected as self-reports from the Basic data set, which had no wave structure. We used the last entry for age and gender in the present analyses.

*Monthly gross income* (in Euro), *working hours* (part-time job vs. full-time job), and *leadership position* (yes vs. no) were drawn from the Employment data set from Wave 8. All measures were self-reported. For working hours, participants were asked whether they worked in full- or part-time employment. Regarding leadership position, participants were asked whether they were leading and/or supervising other employees. In the present analyses, the only income measures we included came from participants’ main job where participants had reported their monthly gross income in Euro from their main job. For participants who did not provide information on their income in Wave 8 but reported that there had been no change in their income since the last assessment, we used the entry from their last valid assessment.

Variables regarding *occupation* were drawn from the Employment data set from Wave 8. Participants reported the precise designation of their current occupation (e.g., geriatric nurse). The NEPS then provides 5-digit codes for each occupation that are equal to the 5-digit code of the classification of occupations by the Federal Agency of Work since 2010 (KldB-2010). This 5-digit code allows a differentiation of occupations in 5 levels (digit 1 describes the affiliation to an occupational sector, digit 2 describes the affiliation to occupational major groups, digit 3 differentiates into occupational groups, digit 4 differentiates subgroups, and digit 5 differentiates professions [69]). The Federal Agency of Work offers registers of all occupations in Germany listed up to the 4-digit code. For example, for the geriatric nurse, the KldB-code according to the Federal Agency of Work would be 8210, as also provided in the Employment data set by the NEPS (see the data manual [70]). We used the classification of occupations provided by the NEPS on the 4-digit level. For participants who did not provide information about their occupation in Wave 8 but reported that their occupation had not changed since the last assessment, we used the entry from their last valid assessment, respectively from their last valid 4-digit code of the classification of occupations (KldB-10) as the cluster variable (N = 484). To derive a measure of the *gender ratio* in occupations, we used the relative frequency of males in occupations that were categorized on the 4-digit level in the present data set.

*Reasoning skills* were assessed via a set of performance tests in the Competence data set in Wave 7. Participants had to complete a matrix test of 12 tasks, where the missing geometrical element for a complete set of figures had to be chosen [71]. The internal consistency (Cronbach’s alpha) in the sample was α = .75.

*Educational attainment* was assessed as participants’ number of years of education. This variable was provided by the NEPS that calculated the years of education by deriving a function based on the classification scheme of the Comparative Analysis of Social Mobility in Industrial Nations (CASMIN) [72]. Therefore, years of education are strongly linked to the level of education but can be handled continuously.

### Analytic strategy

Because of the nested data structure, we used multilevel random coefficient modeling (MRCM [65]) in all analyses. The model codes are provided in the Supporting Information section as S1 and S2 Code. Data preparation was done using R version 3.6.3. [73]. A total of *N* = 6,070 individuals were nested within *N* = 484 occupations.

To test the Theory of Gendered Organizations and to replicate previous findings on gender differences and occupational differences in income, we included income as the dependent variable, gender (men = 0, women = 1) as a predictor at the individual level, and the gender ratio in an occupation (i.e., the relative frequency of men in an occupation) as a predictor at the cluster level. In order to investigate whether employees earned more (or less) in gender-typical than in gender-atypical occupations, we ran moderated multilevel random coefficient modeling, including the interaction term between the gender ratio in an occupation and gender and their respective main effects as predictors.

Furthermore, we included several covariates in the analyses. In addition to gender, research has consistently shown that income depends on working hours, leadership position, educational attainment, and age, all of which are typically controlled for when income differences are tested [9,18]. Employees who are older, work more hours, or hold a leadership position typically earn higher incomes [9]. Women more often work part-time and occupy leadership positions less often than men [13–16]. This is why it is important to control for working hours and leadership position in analyses on the gender pay gap. Gender differences are also present in educational attainment, as women tend to obtain higher degrees than men [2,3]. To avoid such confounds, we also controlled for educational attainment in our analyses. As cognitive ability also predicts career success [74–76] and is not fully covered by educational attainment [77,78], we also included reasoning skills as a proxy for cognitive ability as a covariate.

Thus, in all respective analyses, we controlled for age, reasoning, leadership position (not occupying a leadership position = 0, occupying a leadership position = 1), working hours (part-time = 0, full-time = 1), and years of education at the individual level. To improve the interpretability of the model results [79], all continuous predictor variables at the individual level were group-mean centered so that we could more accurately estimate the variability in the slope between clusters [65,80], and the gender ratio at the cluster level was grand-mean centered [79,80] before we tested the models. All models were run in Mplus Version 7 [81]. Missing data were treated using the model-based full information maximum likelihood (FIML) approach [82]. Finally, we specified all predictors as correlated at the individual and cluster levels to improve the handling of missing values and to reduce bias due to missing data [83]. We defined effects with *p* < .05 as statistically significant.

## Results

Table 1 presents descriptive statistics and zero-order correlations for all variables. Gender was significantly negatively related to income (*r* = -.39), working hours (*r* = -.48), and leadership position (*r* = -.19). If the other variables were not controlled for, women had lower incomes, less often had full-time jobs, and occupied fewer leadership positions than men. The gender ratio in an occupation was positively correlated with income (*r* = .34), such that occupations that tend to be dominated by men had better pay than occupations that tend to be dominated by women.

**Table 1:** Summary of zero-order correlations, means, and standard deviations (n = 6,070).

	Measure	*M*	*SD*	*1*	*2*	*3*	*4*	*5*	*6*	*7*	*8*
1	Income	3.134	2.047	1							
2	Age	0.000	9.083	-.021	1						
3	Reasoning	-0.066	2.190	.108***	-.300***	1					
4	Gender	0.492	0.500	-.391***	-.026*	-.046***	1				
5	Working hours	0.631	0.483	.544***	-.114***	.060***	-.476***	1			
6	Leadership position	0.275	0.449	.367***	.022	-.002	-.194***	.241***	1		
7	Gender ratios in the occupations	0.000	0.316	.336***	.000	-.001	-.633***	.406***	.150***	1	
8	Years of education	-0.001	1.709	.179***	-.137***	.253***	-.049***	.044***	.049***	.000	1

*Note*. Mean income in the present study deviated from mean monthly gross income as assessed by the Federal Office of Statistics (M = 2,857€; [28]). Continuous variables are income (in Euro), age (in years), reasoning (test scores from 0 to 12), the gender ratios in the occupations (ascending ratio of men to women in occupations), and years of education. All continuous variables except income were centered. Dichotomous variables are gender (men = 0, women = 1), working hours (part-time = 0, full-time = 1), and leadership position (no = 0, yes = 1). *M* denotes mean values, *SD* denotes standard deviations.

* p < .05.

** p < .01.

*** p < .001.

### The associations of gender and the gender ratio with income

In order to test the Theory of Gendered Organizations and to replicate previous findings on gender differences and on occupational differences in income, we computed an additive multilevel regression model by including gender, the gender ratios in the occupations, and the covariates as predictors. The results are provided in Table 2.

**Table 2:** Predictors of income: Regression results for the additive MRCM.

Predictor	*B* (95% CI)	SE	P-value
Intercept	2.167 (1.664, 2.669)	0.256	<0.001
Gender ratios in the occupations	0.414 (0.094, 0.735)	0.159	.011
Gender	-0.426 (-0.701, -0.151)	0.140	.002
Years of education	0.167 (0.132, 0.202)	0.018	<0.001
Age	0.009 (-0.001, 0.018)	0.005	.067
Reasoning	0.049 (0.005, 0.093)	0.023	.029
Leadership position	0.677 (0.420, 0.934)	0.131	<0.001
Working hours	1.525 (1.317, 1.734)	0.106	<0.001

*Note*. Variables are coded as follows: income (in Euro), the gender ratios in the occupations (ascending ratio of men in occupations, grand-mean centered), gender (men = 0, women = 1), years of education (in years, group-mean centered), age (in years, group-mean centered), reasoning (test scores from 0 to 12, group-mean centered), leadership position (no = 0, yes = 1), and working hours (part-time = 0, full-time = 1). *B* = unstandardized regression coefficient. The 95% confidence intervals for *B* are presented in parentheses.

The results revealed that a man who worked in an occupation with a relatively equal ratio of men and women (i.e., at the uncentered mean of 51% men), was of average age, did not occupy a leadership position, worked a part-time job, and had average reasoning skills earned an income of 2,167€ per month (*B* = 2.167, *p* < .001). For each additional year of age, an employee was paid 9€ more (*B* = 0.09), however, the regression coefficient for age was not statistically significant (*p* = .067). For each task solved on the reasoning competence test, an employee earned an average of 49€ more (*B* = 0.049, *p* < .05). Occupying a leadership position was associated with 677€ more in income in comparison with not occupying a leadership position (*B* = 0.677, *p* < .001). Furthermore, working a full-time job was associated with 1,525€ more than working a part-time job (*B* = 1.525, *p* < .001), whereas each additional year of education came along with a 167€ increase in income (*B* = 0.167, *p* < .001).

In accordance with previous findings, our analyses revealed that women earned on average 426€ less per month than men did (*B* = -0.426, *p* < .01) even when we controlled for the gender ratio in an occupation, age, reasoning, leadership position, working hours, and years of education.

When we controlled for gender and all the other covariates, the gender ratio in an occupation was significantly associated with income (*B* = 0.414, *p* < .05), meaning that for a 10% increase in the percentage of men in an occupation, a person would earn 41.4€ more per month. Thus, our results are in line with previous findings that showed that occupations with a higher ratio of men pay better than occupations with a lower percentage of male employees.

In addition, we conducted the analyses without the covariates, including only gender and the gender ratio in an occupation as predictors (see S1 Table in the supporting information section). The model code for the additive model without covariates is provided in the supporting information section (S3 Code). Furthermore, we computed a sensitivity analysis. Results are presented in S1 Appendix and S1 Fig.

### The gender pay gap is alive and well–but is moderated by the gender ratio in an occupation

To test whether employees earn more in gender-typical (or gender-atypical) occupations, we computed multilevel random coefficient models that included the cross-level interaction between the gender ratios in the occupations and gender while controlling for age, reasoning, leadership position, years of education, and working hours. The regression results are provided in Table 3.

**Table 3:** Predictors of income: MRCM results for the interaction of gender and the gender ratios in the occupations.

Predictor	*B* (95% CI)	SE	P-value
Intercept	2.180 (1.990, 2.370)	0.097	<0.001
Gender ratios in the occupations	0.115 (-0.356, 0.587)	0.241	0.631
Gender	-0.394 (-0.523, -0.266)	0.066	<0.001
Gender ratios in the Occupations x Gender	0.628 (0.224, 1.032)	0.206	0.002
Years of education	0.167 (0.137, 0.197)	0.015	<0.001
Age	0.009 (0.003, 0.015)	0.003	0.006
Reasoning	0.050 (0.025, 0.074)	0.012	<0.001
Leadership position	0.685 (0.541, 0.828)	0.073	<0.001
Working hours	1.557 (1.391, 1.724)	0.085	<0.001

*Note*. Variables are coded as follows: Income (in Euro), the gender ratios in the occupations (ascending ratio of men in occupations, grand-mean centered), gender (men = 0, women = 1), years of education (in years, group-mean centered), age (in years, group-mean centered), reasoning (test scores from 0 to 12, group-mean centered), leadership position (no = 0, yes = 1), and working hours (part-time = 0, full-time = 1). *B* = unstandardized regression coefficient. The 95% confidence intervals for *B* are presented in parentheses.

**Fig 1:**
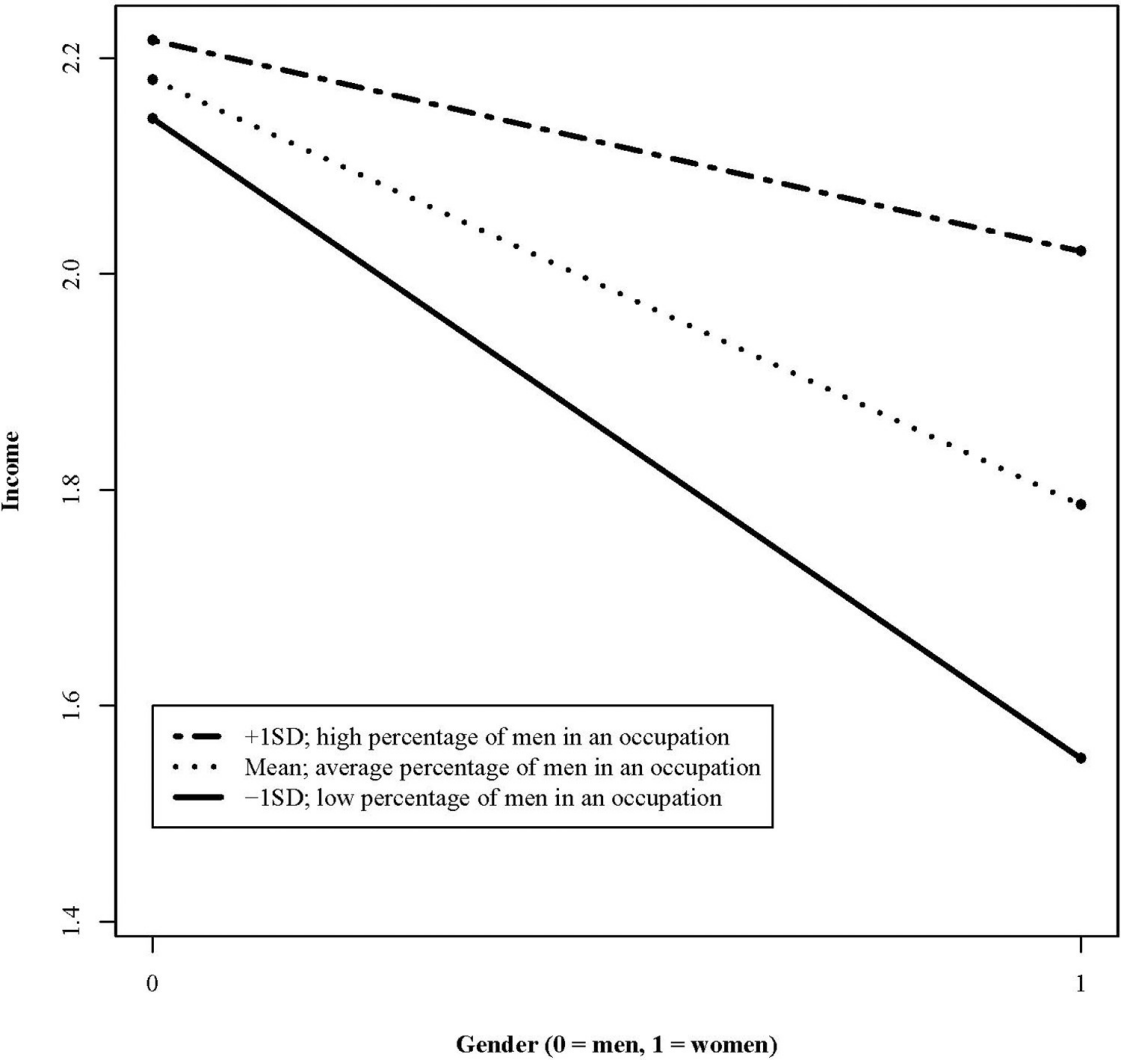
Cross-level-interaction of gender and gender ratio on income with gender ratio as moderator. Simple slopes for men and women in occupations with different gender ratios indicating the relative frequency of men in an occupation. Predicted values in income are in Euro (1 = 1000 Euro) and are based on setting covariates to zero (i.e., men and women of average age, average reasoning skills, average years of education, no leadership position and part-time employment). Values of gender ratio are grand-mean-centered. The figure was created in R.

The interaction between gender and the gender ratio was statistically significant (*B* = 0.628, *p* < .01). To further illustrate the association of gender and income moderated by the gender ratios in the occupations, we computed simple slope tests (see Fig 1) for three values of the gender ratio (-1 standard deviation, 0 standard deviation, + 1 standard deviation above the mean; [84]). Simple slope analyses showed that women earned 592€ less than men working in an occupation with 15% men in an occupation (*B* = -0.592, p < .001). Women who worked in an occupation with a relatively equal ratio of men and women (i.e., 51% of men), earned 394 € less than their male colleagues (*B* = -0.394, p < .001). Women who worked in an occupation with 87% men in an occupation earned 196€ less than men working in the same occupation (*B* = -0.196, p < .001). Results show that the pay gap between men and women is smaller when the ratio of men is higher. The interpretation of the conditional effect of gender ratio on income according to Hayes [64] suggests that working in gender-atypical occupations is linked to income in women, but not in men.

In addition, we computed the analyses without covariates and included only gender and the gender ratio in the occupation as predictors. The direction and significance of the interaction effect between gender and gender ratio remained stable (*B =* 0.559, *p* = .049; for all coefficients, see S2 Table in the supporting information section). The model code for the additive model without the covariates is provided in the Supporting Information section (S4 Code).

## Discussion

We used a recently collected large sample from the German population to test the male advantage in income across occupations (as proposed by the Theory of Gendered Organizations) and whether it varies with the gender ratio in occupations (as proposed by arguments regarding person-job fit and considerations of gender-related visibility that build on the Theory of Tokenism). To test the direction of the relationship between the gender ratio in occupations and the gender pay gap, we proposed two hypotheses concerning the role of the gender ratio in an occupation regarding the gender pay gap by analyzing the interaction between gender and the gender ratio.

### Belonging to the gender minority in an occupation pays for women: The gender pay gap is smaller in occupations with a higher ratio of men

Our results show that the gender pay gap narrows as the ratio of men in an occupation increases. To be more specific, it seems advantageous for women to work in a gender-atypical occupation because as simple slope analyses showed that the negative association of gender and income (as the male advantage) is reduced in occupations with a higher ratio of men (i.e., gender-atypical occupations for women).

Several studies and meta-analyses have consistently shown that, overall, men have higher incomes than women [2,9]. Still, previous studies supporting the Theory of Gendered Organizations have not provided conclusive evidence for the role of the gender ratio in the gender pay gap [18,85]. We tested the Theory of Gendered Organizations across occupations.

Furthermore, we wanted to know whether the magnitude of the male income advantage would vary across occupations that differ with respect to the gender ratio. We tested the direction of an interaction between gender and the gender ratio in an occupation on the basis of arguments about person-job fit [38,39] and gender-related visibility [building on 47,49]. Our results from a large national sample of employees showed that the gender pay gap narrowed in occupations with a higher ratio of men.

Our results did not provide support for the argument that an employee will have an income advantage if the employee’s gender-typical roles and abilities match the occupation´s and the employer´s demands and expectations [38]. Merely belonging to the gender majority and thus being able to set the social standards in an occupation (which might then lead to the advantage of gender-related invisibility [47,86]does not necessarily lead to income advantages.

Previous studies did not find that belonging to the gender minority in an occupation is beneficial for women [17,33,87], however our findings suggest that women can achieve higher incomes in occupations with a higher ratio of men–as shown by a narrowing gender pay gap. Considering men, Judge and colleagues found that men who behaved in a gender-atypical manner had lower income [8]. Our findings suggest that men do not get penalized in terms of income for choosing gender-atypical occupations.

Women can benefit from working in gender-atypical occupations, a trend that may be due to the fact that they are more visible in male-dominated occupations and because this occurs in a context that has recently called for more gender diversity [e.g., 61,88,89]. In such fields, the hiring of women may be on the rise [62], and women may more often be singled out for promotions or bonuses. In line with this reasoning, recent research has shown that highly qualified women in management positions can have higher incomes than their male colleagues in equal positions. In fact, women are often perceived to add value to an organization’s goal to achieve diversity [89].

One reason why our findings differ from previous findings may be the fact that we used a recent and large sample. Our results may thus reflect the changes that have occurred in the last two decades in the zeitgeist regarding higher pressure for gender diversity in occupations [61]. Moreover, we did not analyze only single occupational fields as some previous studies did [53,55]. Rather, we provided an overview across a variety of occupations and did not categorize occupations but used a continuous approach.

Results also showed that gender differences in income were particularly pronounced in predominantly female occupations and declined as the ratio of men in an occupation increased. A recent a meta-analysis on gender differences in performance and rewards stated the opposing trend, namely, that the gender pay gap narrows in occupations with a decreasing ratio of men [90]. The differences may result from different labor markets analyzed, as the meta-analysis used samples from the United States and our study relied on a German sample. For example in Germany, income and working conditions are partly negotiated between union representatives and employers and there is less individual divergence. In line with this, the OECD stated for 2016 that in Germany the percentage of collective bargaining coverage was 56.0%, whereas for example in the United States it was only 11.5% [91]. In accordance with this reasoning, the gender pay gap is smaller in the public service sector (where collective wage agreements are common) than in the private industry [92]. Recent figures provided by the Federal Statistical Office support our result of the narrowing gender pay gap in male-dominated occupations: In 2020, the gender pay gap was the largest in the fields of art and entertainment, service providers and health care, whilst it narrowed in public service, hospitality industry, transport, mining industry and raw material extraction [92].

Our findings show that for women, it can be beneficial in terms of income to work in a gender-atypical occupation. This benefit may come from gender-related visibility and the rising demands for gender equality in the workplace. Still, there are persisting career barriers for women that result from gender stereotypes and discrimination that prevent the pay gap from closing [93,94].

### Income differences are still in favor of men

Another important finding of the present study is that men still earn more than women across occupations. The result is in line with previous findings [18] and may point to an underappreciation of women´s skills and traits in the labor market [4,95,96]. Stereotypical perceptions of women with respect to communal traits such as being emotional, empathic, and considerate and men as possessing agentic traits such as being assertive, decisive, and competitive [97,98] may contribute to a lack of appreciating women because agentic traits are typically perceived as beneficial for career success [99].

### Occupations with a higher ratio of men pay best

Our analyses indicated that occupations with a higher ratio of men overall pay best even when controlling for the gender of the respective employee–and that remains true when blue-collar occupations are included. This finding supports the status composition hypothesis and implies that there is discrimination in certain occupations in terms of income. This discrimination may be based on the attributes of the majority of the employees [30] in that occupation (in our case, the majority gender, but in other cases, the majority ethnicity, etc.). In this vein, stereotypical beliefs regarding women´s traits and abilities may be reflected in the devaluation of predominantly female occupations [96].

### Limitations and directions for future studies

While offering a new perspective on income differences between men and women with a large recent sample, our study does have some limitations. Although we interpreted our findings inter alia as reflecting gender-related visibility, our interpretations are limited because we did not directly assess gender-related visibility, but we assumed that the finding was due to the kind of visibility that arises from the gender ratios in occupations. Gender-related visibility refers to the fact that an employee who works in a gender-atypical occupation stands out on the basis of gender [47]. Future research could assess effects of perceived gender-related visibility by employers on the basis of field studies to provide more insight into this assumption. Beyond this, effects on income differences in other types of visibility (e.g., reflected in ethnicity) could be investigated.

Future studies are needed to verify the robustness of our findings. When we analyzed the models without the covariates, the interaction between gender and the gender ratios in the occupations just barely reached the level of significance. Future research might want to retain the continuous approach of operationalizing the gender ratios in occupations with population-relevant samples but use an even larger sample size at the individual and cluster levels. According to our sensitivity analysis, larger sample sizes, especially at the cluster level, will also be helpful for improving the statistical power to detect the interaction between gender and the gender ratios. In multilevel designs, increasing the sample size at the cluster level is more important for the power than increasing the sample size at the individual level [for an overview of simulation research, see 100]. Thus, larger samples with a sufficiently high cluster sample size can help to test the robustness of the present findings regarding the relevance of the gender ratio in an occupation for the gender pay gap.

Furthermore, we controlled for only a limited set of variables in the analyses. Future investigations might include additional potentially relevant variables, such as motivation and ambition [101], the desire for mobility [102], and career interruptions due to factors such as parental leave [6].

Gender differences in preferences for occupational-specific traits might also be worth examining to explain gender segregation and the gender pay gap across occupations. For example, Cortes and Pan [103] showed that women were more often found in occupations that were less competitive, placed greater emphasis on social contributions (as compared with success and money), were more flexible, and required more interactional and less physical skills [103]. Furthermore, Levanon and Grusky [104] painted a fine-grained picture of how segregation works in favor of or against women and men by differentiating occupation-specific traits. Their results with recent U.S. census data indicated that essentialism (i.e., beliefs about gender-specific interests and skills) accounts for a large proportion of segregation [104]. Specifically, their results showed that the form of interactional essentialism (e.g., sociability-requiring occupations) is disadvantageous for women in terms of income. Whereas the effects of analytical essentialism (i.e., the extent to which an occupation requires problem-solving or mathematical skills) were weak, and men had only a small advantage, physical essentialism was the only form of essentialism in favor of women: Occupations that required manual work and physical strength were low in pay and strongly frequented by men [104]. This research uncovers the detailed mechanisms of how gender segregation in occupations comes about and how occupational traits can help to uncover it.

Unfortunately, to our knowledge, there is not yet a German equivalent to the categorization of occupational traits as offered in the U.S. occupational data archive O*NET [used by 104]. However, future research could try to incorporate occupational characteristics to explain the gender pay gap by applying a similar approach to research by Denissen et al. [105], who had two independent occupational experts from the German Federal Employment Agency rate the personality demands of occupations in their sample to provide a measure of occupational traits.

Recent research has also focused on another set of variables to explain gender segregation in the labor market. Working with the Dictionary of Occupational Titles (DOT), U.S. census data, and data from the American Community Survey, Baker and Cornelson [106] showed how gender differences in sensory functions, perceptual motor tasks, and visuospatial skills contribute to occupational gender segregation in the labor market (e.g., men’s ability to better tolerate noise leads to strong gender segregation in noisy occupations). Future research might want to investigate gender differences in sensory, motor, and spatial skills and how the resulting gender segregation relates to the gender pay gap.

Finally, it seems worthwhile to investigate how much agency and communion is reflected in occupational characteristics. Agency includes competence, assertiveness, and decisiveness, whereas communion includes helpfulness, benevolence, and trustworthiness [97]. Representative data from the US on gender stereotypes has shown that over time, beliefs about gender differences in competence have changed in favor of women, and the female advantage in communion has increased, whereas the male advantage in agency has not changed [107]. Still, stereotypical beliefs about the prevalence of these traits in women and men [34,35] prevail, and the preference for agency in high-paying positions, such as in management or on executive boards [37], seems persistent [2,36]. Even though, over time, beliefs about leadership qualities seem to have incorporated communal traits and thus might reflect a change in the appreciation of female leadership styles, the perceived need for agentic traits for successful leadership still prevails [36]. Thus, future research could investigate whether beliefs about these traits are reflected at an organizational level and whether they have an effect on the gender pay gap.

The present results replicated findings on the male advantage in income but extended this evidence by also showing support for the Theory of Tokenism. Our findings imply that the wage gap narrows as the ratio of men to women in an occupation increases. In other words, in male-dominated occupations, women might have a relative economic advantage that might be attributed to increased gender-related visibility or the rising demand for gender diversity in male-dominated occupations. However, as our analyses were cross-sectional, our study does not allow us to draw causal conclusions. Future studies may benefit from employing longitudinal designs to test the robustness of findings and experimental designs that use direct measures of gender-related visibility. Also, we cannot draw conclusions for work cultures and labor markets that are not experiencing an increase in the emphasis on gender diversity in occupations.

Not only might future studies wish to focus on uncovering additional factors in the adjusted gender pay gap, but they could also investigate the factors that are known to be responsible for the gender pay gap even further, for example, by analyzing factors that influence decisions to work full-time as well as differences in opportunities for a full-time job across predominantly male or female occupations. Finally, as the International Labour Organization (ILO) observed recently, women are more often involved in unpaid work (e.g., care work) than men [108] even in two-income households [109], which may be linked to time constraints, gender differences in career choices, and a lack of opportunities to engage in full-time work for women. Future research is needed to disentangle the complex interplay of these factors.

## Supporting information

S1 Fig Plot for the sensitivity analysis. *Note*. The plot shows the sensitivity of the random coefficient model to detect a significant effect. The horizontal blue line represents a power of 80%, the horizontal red line represents a power of 95%. The figure was created in R.
(TIF)

S1 Table Predictors of income: Regression results for the additive MRCM with and without covariates. *Note*. Variables are coded as follows: income (in Euro), gender ratio in occupations (ascending ratio of men in occupations, grand-mean centered), gender (men = 0, women = 1), years of education (in years, group-mean centered), age (in years, group-mean centered), reasoning (test scores from 0 to 12, group-mean centered), leadership position (no = 0, yes = 1), and working hours (part-time = 0, full-time = 1). *B* = unstandardized regression coefficient, CI = 95% confidence interval. The sample size was *N =* 6,070 for the model with and without covariates respectively.
(DOCX)

S2 Table Predictors of income: MRCM results for the interaction of gender and the gender ratio in occupations with and without covariates. *Note*. Variables are coded as follows: income (in Euro), gender ratio in occupations (ascending ratio of men in occupations, grand-mean centered), gender (men = 0, women = 1), years of education (in years, group-mean centered), age (in years, group-mean centered), reasoning (test scores from 0 to 12, group-mean centered), leadership position (no = 0, yes = 1), and working hours (part-time = 0, full-time = 1). *B* = unstandardized regression coefficient, CI = 95% confidence interval. The sample size was *N =* 6,070 for the model with and without covariates respectively.
(DOCX)

S1 Appendix Sensitivity analysis. (DOCX)

S1 Code Mplus model code for the additive MRCM. The German short variable names used in the model are “reason” for *reasoning*, “Alter” for *age*, “Stelle” for *working hours*, “Hierarchie” for *leadership position*, “bjahre” for *years of education*, “Gender” for *gender*, “Gen4_ratio” for *gender ratio in occupations*, and “Gehal_t” for *income*.
(PDF)

S2 Code Mplus model code for the cross-level-interaction MRCM. The German short variable names used in the model are “reason” for *reasoning*, “Alter” for *age*, “Stelle” for *working hours*, “Hierarchie” for *leadership position*, “bjahre” for *years of education*, “Gender” for *gender*, “Gen4_ratio” for *gender ratio in occupations*, and “Gehal_t” for *income*.
(PDF)

S3 Code Mplus model code for the additive MRCM without covariates. The German short variable names used in the model are “Gender” for *gender*, “Gen4_ratio” for *gender ratio in occupations*, and “Gehal_t” for *income*.
(PDF)

S4 Code Mplus model code for the cross-level-interaction MRCM without covariates. The German short variable names used in the model are “Gender” for *gender*, “Gen4_ratio” for *gender ratio in occupations*, and “Gehal_t” for *income*.
(PDF)
