# Supplementary figures and images for: The gender pay gap is smaller in occupations with a higher ratio of men: Evidence from a national panel study
Source: PLoS One. 2022 Jul 6;17(7):e0270343. doi: 10.1371/journal.pone.0270343 (PMC9258844; doi:10.1371/journal.pone.0270343)

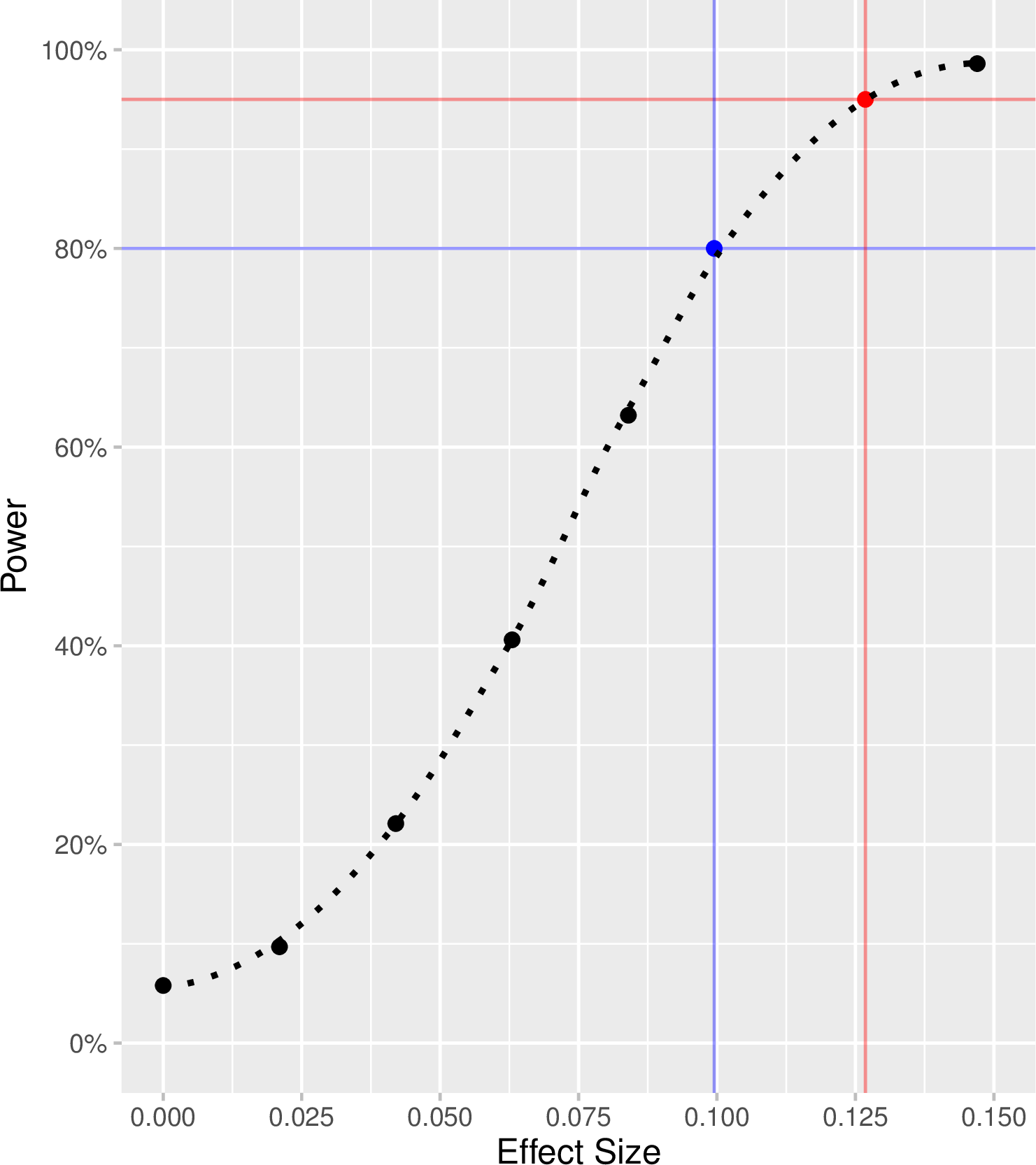

Supplement: S1 Fig — Note. The plot shows the sensitivity of the random coefficient model to detect a significant effect. The horizontal blue line represents a power of 80%, the horizontal red line represents a power of 95%. The figure was created in R. (TIF) [file pone.0270343.s001.tif]
