# Supplementary material for: The gender pay gap is smaller in occupations with a higher ratio of men: Evidence from a national panel study
Source: PLoS One. 2022 Jul 6;17(7):e0270343. doi: 10.1371/journal.pone.0270343 (PMC9258844; doi:10.1371/journal.pone.0270343)
Supplement: S1 Table — Note. Variables are coded as follows: income (in Euro), gender ratio in occupations (ascending ratio of men in occupations, grand-mean centered), gender (men = 0, women = 1), years of education (in years, group-mean centered), age (in years, group-mean centered), reasoning (test scores from 0 to 12, group-mean centered), leadership position (no = 0, yes = 1), and working hours (part-time = 0, full-time = 1). B = unstandardized regression coefficient, CI = 95% confidence interval. The sample size was N = 6,070 for the model with and without covariates respectively. (DOCX) [file pone.0270343.s002.docx]

**S1 Table. Predictors of income: Regression results for the additive MRCM with and without covariates**

| **Predictor** | Without covariates | | | With covariates | | |
| --- | --- | --- | --- | --- | --- | --- |
|  | **B (95% CI)** | **SE** | **P-value** | **B (95% CI)** | **SE** | **P-value** |
| Intercept | 3.670 (3.515, 3.825) | 0.079 | <0.001 | 2.167 (1.664, 2.669) | 0.256 | <0.001 |
| Gender ratio in occupations | 0.771 (0.417, 1.125) | 0.181 | <0.001 | 0.414 (0.094, 0.735) | 0.159 | .011 |
| Gender | -1.106 (-1.255, -0.957) | 0.076 | <0.001 | -0.426 (-0.701, -0.151) | 0.140 | .002 |
| Years of education |  |  |  | 0.167 (0.132, 0.202) | 0.018 | <0.001 |
| Age |  |  |  | 0.009 (-0.001, 0.018) | 0.005 | .067 |
| Reasoning |  |  |  | 0.049 (0.005, 0.093) | 0.023 | .029 |
| Leadership position |  |  |  | 0.677 (0.420, 0.934) | 0.131 | <0.001 |
| Working hours |  |  |  | 1.525 (1.317, 1.734) | 0.106 | <0.001 |

*Note.* Variables are coded as follows: income (in Euro), gender ratio in occupations (ascending ratio of men in occupations, grand-mean-centered), gender (men = 0, women = 1), years of education (in years, group-mean-centered), age (in years, group-mean-centered), reasoning (test scores from 0 – 12, group-mean-centered), leadership position (no = 0, yes = 1), and working hours (part-time = 0, full-time = 1). B = unstandardized regression coefficient, CI = 95% confidence interval. The sample size was *N =* 6,070 for the model with and without covariates respectively.
