# Supplementary material for: The gender pay gap is smaller in occupations with a higher ratio of men: Evidence from a national panel study
Source: PLoS One. 2022 Jul 6;17(7):e0270343. doi: 10.1371/journal.pone.0270343 (PMC9258844; doi:10.1371/journal.pone.0270343)
Supplement: S2 Table — Note. Variables are coded as follows: income (in Euro), gender ratio in occupations (ascending ratio of men in occupations, grand-mean centered), gender (men = 0, women = 1), years of education (in years, group-mean centered), age (in years, group-mean centered), reasoning (test scores from 0 to 12, group-mean centered), leadership position (no = 0, yes = 1), and working hours (part-time = 0, full-time = 1). B = unstandardized regression coefficient, CI = 95% confidence interval. The sample size was N = 6,070 for the model with and without covariates respectively. (DOCX) [file pone.0270343.s003.docx]

**S2 Table. Predictors of income: MRCM results for the interaction of gender and gender ratio in occupations with and without covariates**

| **Predictor** | Without covariates | | | With covariates | | |
| --- | --- | --- | --- | --- | --- | --- |
|  | **B (95% CI)** | **SE** | **P-value** | **B (95% CI)** | **SE** | **P-value** |
| Intercept | 3.750 (3.573, 3.928) | 0.091 | <0.001 | 2.180 (1.990, 2.370) | 0.097 | <0.001 |
| Gender ratio in occupations | 0.429 (-0.057, 0.915) | 0.248 | 0.083 | 0.115 (-0.356, 0.587) | 0.241 | 0.631 |
| Gender | -1.125 (-1.279, -0.971) | 0.078 | <0.001 | -0.394 (-0.523, -0.266) | 0.066 | <0.001 |
| Gender ratio in occupations x Gender | 0.559 (0.002, 1.115) | 0.284 | 0.049 | 0.628 (0.224, 1.032) | 0.206 | 0.002 |
| Years of education |  |  |  | 0.167 (0.137, 0.197) | 0.015 | <0.001 |
| Age |  |  |  | 0.009 (0.003, 0.015) | 0.003 | 0.006 |
| Reasoning |  |  |  | 0.050 (0.025, 0.074) | 0.012 | <0.001 |
| Leadership position |  |  |  | 0.685 (0.541, 0.828) | 0.073 | <0.001 |
| Working hours |  |  |  | 1.557 (1.391, 1.724) | 0.085 | <0.001 |

*Note.* Variables are coded as follows: income (in Euro), gender ratio in occupations (ascending ratio of men in occupations, grand-mean-centered), gender (men = 0, women = 1), years of education (in years, group-mean-centered), age (in years, group-mean-centered), reasoning (test scores from 0 – 12, group-mean-centered), leadership position (no = 0, yes = 1), and working hours (part-time = 0, full-time = 1). B = unstandardized regression coefficient, CI = 95% confidence interval. The sample size was *N =* 6,070 for the model with and without covariates respectively.
