# Supplementary material for: The gender pay gap is smaller in occupations with a higher ratio of men: Evidence from a national panel study
Source: PLoS One. 2022 Jul 6;17(7):e0270343. doi: 10.1371/journal.pone.0270343 (PMC9258844; doi:10.1371/journal.pone.0270343)
Supplement: S1 Appendix — (DOCX) [file pone.0270343.s004.docx]

# S1 Appendix. Sensitivity analysis

According to Bartlett [1], a sensitivity power analysis identifies the effect sizes a test is sensitive enough to detect given the sample size and the power. We computed a sensitivity analysis with standardized variables in R [2] with the simr package [3] using simulation methods [e.g., 4]. We ran a sensitivity analysis for an individual sample size of 6,070, a cluster sample size of 484, and a significance level of α = .05 at a power of 80% and at a power of 95%. The results showed that the present study was able to detect a minimum standardized cross-level interaction effect of β = 0.0995 with 80% power. With a power of 95%, the study was a able to detect a minimum standardized cross-level interaction effect of β = 0.1268. The standardized cross-level interaction effect in our original analyses was β *=* 0.083. We also report the results of the sensitivity analysis as Supporting Information in Figure S1.

References

1. Bartlett, James, E. Introduction to Power Analysis: A Guide to G*Power, jamovi, and Superpower; 2022. Available from: URL: https://osf.io/zqphw/.

2. R: A language and environment for statistical computing. Version 3.6.3. Vienna, Austria: R Foundation for Statistical Computing; 2020. Available from: URL: https://www.R-project.org/.

3. SIMR: an R package for power analysis of generalized linear mixed models by simulation; 2016.

4. DeBruine LM, Barr DJ. Understanding mixed effects models through data simulation [cited 2022 Apr 9]. Available from: URL: https://debruine.github.io/lmem_sim/articles/paper.html.
