# Supplementary material for: The gender pay gap is smaller in occupations with a higher ratio of men: Evidence from a national panel study
Source: PLoS One. 2022 Jul 6;17(7):e0270343. doi: 10.1371/journal.pone.0270343 (PMC9258844; doi:10.1371/journal.pone.0270343)
Supplement: S1 Code — The German short variable names used in the model are “reason” for reasoning, “Alter” for age, “Stelle” for working hours, “Hierarchie” for leadership position, “bjahre” for years of education, “Gender” for gender, “Gen4_ratio” for gender ratio in occupations, and “Gehal_t” for income. (PDF) [file pone.0270343.s005.pdf]

```
DATA:
FILE = "U:/Paper Occupations_multilevel/Bildungsjahre/df_occ_mplus_bjahre_katrin.dat";
define: Gehal_t = Gehaltw8*0.001;
center reason Alter bjahre (GROUPMEAN);
center Gen4_ratio(GRANDMEAN);

 VARIABLE:
 NAMES = kldb4 kldb3 kldb2 kldb5 Berufkat ID_t Alter Gender isced casmin bjahre
     reason kldb Genderberuf Gehaltw8 Hierarchie Stelle At_BA Gen_ratio
     Gen_ratio_zen Alter_zen reason_zen Gen5_ratio Gen4_ratio Gen3_ratio Gen2_ratio
     Gen2_ratio_cgm Gen4_ratio_cgm Gen3_ratio_cgm Alter_gm2 Alter_cwc2 reason_gm2
     reason_cwc2 Alter_gm3 Alter_cwc3 reason_gm3 reason_cwc3 Alter_gm4 Alter_cwc4
     reason_gm4 reason_cwc4;



USEVARIABLES = reason Alter Stelle Hierarchie Gender Gen4_ratio bjahre Gehal_t;

MISSING=all(-999);

cluster = kldb4;



WITHIN = reason Alter Stelle Hierarchie Gender bjahre;

BETWEEN = Gen4_ratio;

ANALYSIS:
type = twolevel random;
algorithm = integration;
```

```
integration = montecarlo;


MODEL:
%within%
Beta1j | Gehal_t ON Gender;
Beta2j | Gehal_t ON reason;
Beta3j | Gehal_t ON Alter;
Beta4j | Gehal_t ON Stelle;
Beta5j | Gehal_t ON Hierarchie;
Beta6j | Gehal_t ON bjahre;



reason WITH Alter Stelle Hierarchie Gender bjahre;
Alter WITH Stelle Hierarchie Gender bjahre;
Stelle WITH Hierarchie Gender bjahre;
Hierarchie WITH Gender bjahre;
Gender WITH bjahre;

%between%
Gehal_t ON Gen4_ratio;

Beta1j WITH Gehal_t;

OUTPUT:
SAMPSTAT TECH1 TECH4 CINTERVAL;
```
