# Supplementary material for: The gender pay gap is smaller in occupations with a higher ratio of men: Evidence from a national panel study
Source: PLoS One. 2022 Jul 6;17(7):e0270343. doi: 10.1371/journal.pone.0270343 (PMC9258844; doi:10.1371/journal.pone.0270343)
Supplement: S4 Code — The German short variable names used in the model are “Gender” for gender, “Gen4_ratio” for gender ratio in occupations, and “Gehal_t” for income. (PDF) [file pone.0270343.s008.pdf]

```
DATA:

FILE = "C:/Users/Administrator/Desktop/noc/df_occ_mplus_bjahre_katrin.dat";

define: Gehal_t = Gehaltw8*0.001;

center reason Alter bjahre (GROUPMEAN);

center Gen4_ratio(GRANDMEAN);


 VARIABLE:

 NAMES = kldb4 kldb3 kldb2 kldb5 Berufkat ID_t Alter Gender isced casmin bjahre

     reason kldb Genderberuf Gehaltw8 Hierarchie Stelle At_BA Gen_ratio

     Gen_ratio_zen Alter_zen reason_zen Gen5_ratio Gen4_ratio Gen3_ratio
Gen2_ratio

     Gen2_ratio_cgm Gen4_ratio_cgm Gen3_ratio_cgm Alter_gm2 Alter_cwc2
reason_gm2

     reason_cwc2 Alter_gm3 Alter_cwc3 reason_gm3 reason_cwc3 Alter_gm4
Alter_cwc4

     reason_gm4 reason_cwc4;




USEVARIABLES = reason Alter Stelle Hierarchie Gender Gen4_ratio bjahre
Gehal_t;


MISSING=all(-999);


cluster = kldb4;
```

```
WITHIN = reason Alter Stelle Hierarchie Gender bjahre;

BETWEEN = Gen4_ratio;

ANALYSIS:
type = twolevel random;
algorithm = integration;
integration = montecarlo;

MODEL:
%within%
Beta1j | Gehal_t ON Gender;



reason WITH Alter Stelle Hierarchie Gender bjahre;
Alter WITH Stelle Hierarchie Gender bjahre;
Stelle WITH Hierarchie Gender bjahre;
Hierarchie WITH Gender bjahre;
Gender WITH bjahre;

%between%
```

```
Gehal_t ON Gen4_ratio;

Beta1j ON Gen4_ratio;

Beta1j WITH Gehal_t;


OUTPUT:

SAMPSTAT TECH1 TECH3 CINTERVAL;
```
